# Inferring the Abundance of an Island Megaherbivore From Semi‐Structured Distance Sampling

**DOI:** 10.1002/ece3.74282

**Published:** 2026-09-04

**Authors:** Lara B. Hillyard, Nancy Bunbury, Christopher N. Kaiser‐Bunbury, Dave Hodgson, Luke A'Bear, Cheryl L. Sanchez, Christopher W. Jones, April Burt, Frauke Fleischer‐Dogley, Dave W. Hudson

**Affiliations:** ^1^ Centre for Ecology and Conservation University of Exeter, Cornwall Campus Penryn UK; ^2^ Seychelles Islands Foundation Victoria Mahé Seychelles

**Keywords:** Aldabra giant tortoise, Bayesian inference, density dynamics, distance sampling, population estimate, Seychelles, state‐space modelling

## Abstract

A core component of conservation management projects is the estimation and monitoring of abundance of target species, in space and through time. Monitoring wildlife is complicated and relies on detection, which itself depends on environmental conditions, phenotypes and behaviours of the species being monitored. Aldabra giant tortoises ( *Aldabrachelys gigantea* ) have great importance as ecosystem engineers, keystone species and ecological replacements. Accurate monitoring is vital to understanding both the size and dynamics of their native and translocated populations, and the species' influence on the wider ecosystem. Despite a long history of organised research and population surveys in their native range on Aldabra Atoll in the Seychelles, to date no rigorous, atoll‐wide estimation of giant tortoise population size has been attempted. The complexity of irregular transect surveys, through a variety of habitats, in several areas of the atoll, necessitates the use of state‐space statistical models that capture heterogeneities in both abundance and detection. Here we develop a Bayesian Hierarchical Distance Sampling (HDS) model to infer habitat‐ and season‐dependent detectability and abundance of the Aldabra giant tortoise population on Aldabra. Between 2018 and 2021 the population was apparently relatively stable and numbered approximately 180,000 individuals, the highest estimate so far made for this population. We attribute this stability and high abundance to the extended history of conservation efforts on Aldabra, including invasive alien species eradication which should be maintained and expanded. We provide a reproducible workflow for Bayesian distance sampling methods using data‐augmentation and habitat‐dependent detectability and recommend its use on legacy datasets and future abundance surveys for similar species. These methods enable robust inference of abundance and detectability even when data are irregular or inconsistently collected, providing a framework for integrating legacy datasets with modern conservation workflows.

## Graphical abstract

We use a hierarchical Bayesian distance‐sampling model to infer the atoll‐wide abundance and habitat‐specific density of Aldabra giant tortoises from long‐term, irregular transect monitoring on Aldabra Atoll (Seychelles). The detection‐corrected wet‐season estimate indicates a large, stable population of ~182,500 individuals (2018–2021), supporting the long‐term success of conservation management and providing a practical template for extracting robust population estimates from imperfect monitoring data.

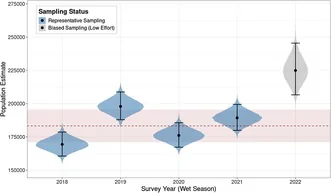

**Abbreviations**
HDS: hierarchical distance sampling
IUCN: International Union for the Conservation of Nature
SIF: Seychelles Islands Foundation; UNESCO; United Nations Educational, Scientific and Cultural Organisation

## 1 Introduction

Obtaining reliable estimates of wild and managed population sizes and dynamics is challenging, as habitat complexity and environmental factors can greatly affect detectability, leading to potential measurement error, bias, and incomplete datasets (Buckland et al. 2023; Jones 2011; Sollmann et al. 2013; Zwerts et al. 2021). Yet without credible measures of abundance through time, it is difficult to measure and monitor the effectiveness of conservation interventions (Nichols and Williams 2006). Robust sampling methods and appropriate analytical techniques are essential to reduce uncertainty and improve data quality. Repeated population sampling can reveal seasonal dynamics and track population changes over time (Lou and Zhao 2017; Mizuki et al. 2020; Sollmann et al. 2013). Integrating environmental covariates with sampling data provides insight into the drivers of population change, supporting the development of effective conservation strategies. However, visual encounter surveys, commonly used in wild population studies, often underestimate population size due to observer bias and variation in detectability among habitats (Fragoso et al. 2019; Newson et al. 2008; Walker 2012).

Distance sampling addresses the challenge of variable detection probability by modelling the likelihood of observing individuals at different distances from a transect (Buckland et al. 2001; Thomas et al. 2010). While distance sampling can provide more accurate population density estimates than raw counts (Swann et al. 2002; Thomas et al. 2010), standard methods can struggle to accurately estimate model parameters when sampling effort is irregular and due to the inherent uncertainty of ‘missing’ individuals. The Bayesian Hierarchical Distance Sampling (HDS) framework (Royle et al. 2004) addresses these challenges by integrating the observation and abundance processes into a single hierarchical model. This structure allows for ‘information sharing’ across transect sections which stabilises estimates in data‐sparse habitats. Unobserved individuals can be accounted for using a technique known as data augmentation, where the model estimates how many individuals may have been present but not detected during surveys. These approaches allow uncertainty in detection and availability to propagate into the final population estimates (Kéry and Royle 2016). Consequently, Bayesian HDS provides a robust tool for analysing complex ecological systems where detection and abundance are inherently linked (Clark et al. 2005; Royle and Dorazio 2008, 2009).

The world's remaining giant tortoises comprise the Aldabra giant tortoise ( *Aldabrachelys gigantea* ) native to Aldabra Atoll in the Seychelles, and the Galápagos giant tortoise (*Chelonoidis niger*) complex which comprises 13 living subspecies on the Galápagos Islands (Gaughran et al. 2025). The Aldabra giant tortoise has the largest free‐living population, with an estimated 100,000 tortoises present on Aldabra in the 1990s (Bourn et al. 1999), following significant exploitation in the 1800s which had reduced the population to less than a thousand individuals (Stoddart and Peake 1979). Aldabra giant tortoises are crucial to the atoll's ecosystem, acting as a flagship species for conservation efforts, a keystone megaherbivore species and an ecosystem engineer that shapes terrestrial habitats (Walton et al. 2019). The species has been monitored on Aldabra since 1979, with the most recent population estimate in 1999 (Bourn et al. 1999), yet definitive population evaluations using modern techniques are lacking. Up‐to‐date and accurate demographic analysis provides a baseline for monitoring population health and dynamics and, despite historical population growth, climatic extremes and habitat change could alter future trajectories; hence, regular design‐based monitoring remains vital.

On Aldabra, the giant tortoise still faces multiple threats. Increasing drought periods and potential sea level rise caused by climate change are predicted to impact the tortoise population by reducing shade and forage (Haverkamp et al. 2017). Invasive alien mammals have also historically affected the behaviour and survivorship of the species (Swingland and Coe 1979), particularly feral goats, which reduced the availability of shade and food resources through overbrowsing (Bourn et al. 1999; Coblentz and Vuren 1987). Feral goats were eradicated from Aldabra in 2012 (Bunbury et al. 2018), but feral cats and black rats remain across much of the atoll, and both are known to consume tortoise eggs and hatchlings (IUCN 2020).

Aldabra giant tortoises hold significant international conservation value and are increasingly used as ‘ecological analogue species’ in rewilding projects on islands in the Indian Ocean (e.g., in Mauritius—Griffiths et al. 2011; Rodrigues—Hansen 2015; and Seychelles—Hansen et al. 2010) to restore the ecological roles once filled by recently extinct megaherbivores (Castellano et al. 2013; Falcón and Hansen 2018; Joseph et al. 2024; Moorhouse‐Gann et al. 2022). For example, a rewilding project on Ile aux Aigrettes off the coast of Mauritius leveraged the tortoises' role as seed dispersers to successfully boost the population of the critically endangered ebony tree (Griffiths et al. 2011). To ensure the long‐term success of these rewilding efforts, understanding the underlying ecological processes and dynamics of the species in its native habitat is vital as this knowledge helps sustain the ecosystem functions they provide (Falcón and Hansen 2018; Griffiths et al. 2011). Establishing effective monitoring methods for the original Aldabra giant tortoise population, and by extension, the translocated populations, allows conservationists to develop mitigation measures that have the potential to strengthen these ecosystems against future climate change (Falcón and Hansen 2018). Aldabra giant tortoises show foraging preferences, favouring leaves, grasses, and woody plants, and they seek shade to prevent overheating (Swingland and Frazier 1980; Walton et al. 2019). These behaviours impact detection, as tortoises may be concealed in vegetation or shaded areas thereby affecting population estimates for both native and managed populations (Falcón et al. 2018; Merton et al. 1976). Addressing these detection challenges by incorporating environmental covariates can improve survey accuracy and provide conservationists with a clearer picture of population health, supporting ongoing and future rewilding efforts.

Here, we adopt the Bayesian HDS approach to abundance‐estimation of Aldabra giant tortoises in their native range on Aldabra, to account for heterogeneity in detection probability arising from habitat variation and to characterise uncertainties associated with missing information within the dataset (Daniels and Hogan 2008; Marques and Buckland 2003; Newson et al. 2008). We address the issue of unobserved individuals by explicitly modelling habitat‐dependent detectability and using data augmentation to represent unobserved individuals. Our aims are to (1) generate an up‐to‐date, credible estimate of the Aldabra giant tortoise population size and density; and (2) demonstrate the power of the Bayesian approach combined with data augmentation when analysing imperfect field observation data, with benefits for maintaining managed tortoise populations used in restoration projects.

## 2 Materials and Methods

### 2.1 Study Site

**FIGURE 1:**
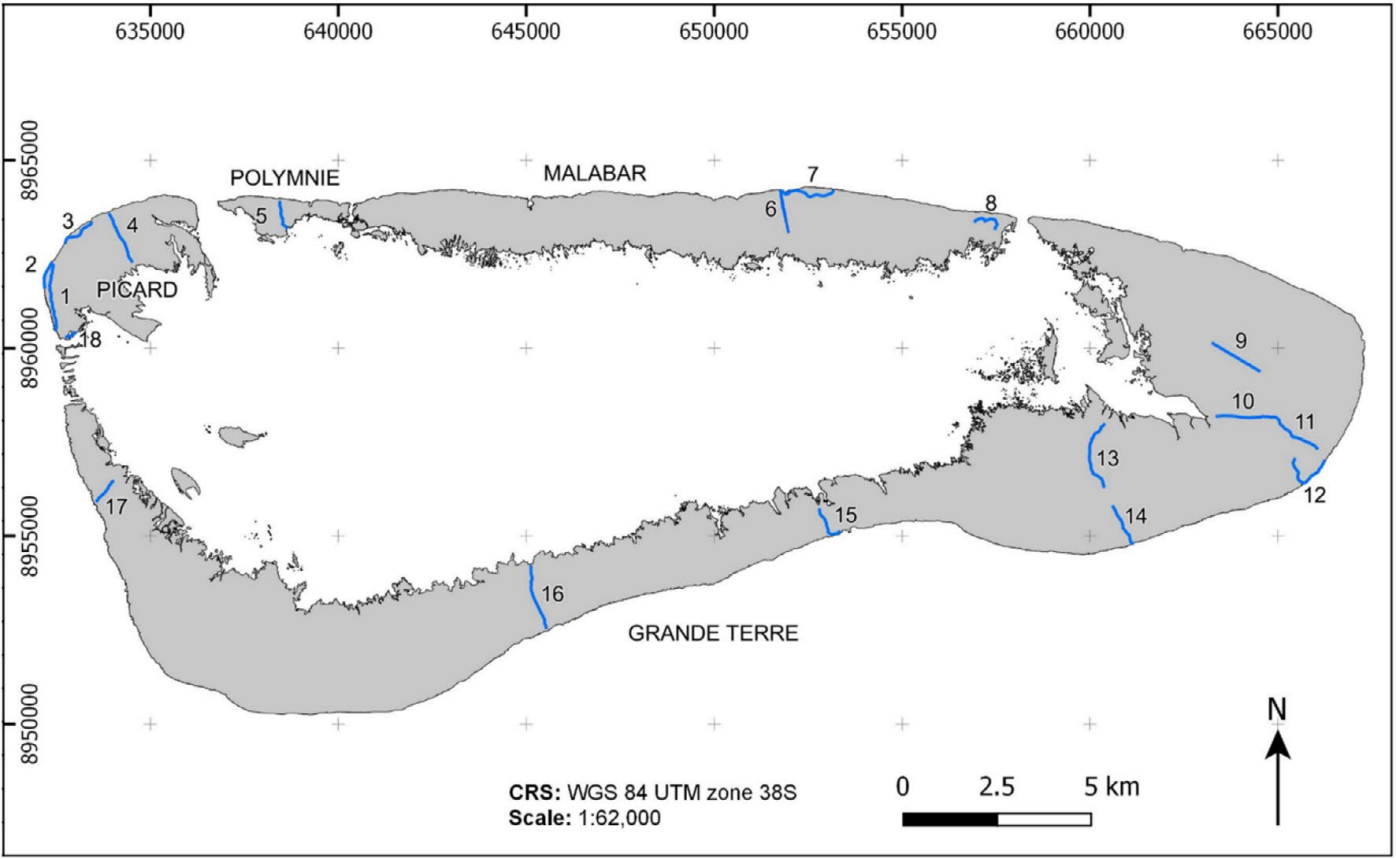
Aldabra Atoll, showing the four main islands and the tortoise transects monitored from 2017 to 2022 (in blue; 1–2—Picard; 3–4—Anse Var; 5—Polymnie; 6–7—Malabar; 8—Middle Camp; 9–12—Cinq Cases; 13–14—Takamaka; 15—Dune Jean‐Louis; 16—Dune d'Messe; 17—Anse Mais; 18—La Gigi).

Aldabra (9°24′ S, 46°20′ E) is one of the world's largest raised coral atolls and is part of the Outer Islands of the Seychelles, in the Western Indian Ocean. The atoll is comprised of four main islands: Picard, Grande Terre, Malabar, and Polymnie, which cover a total land area of 155.5 km^2^ at an average height of ~8 m above sea level (Figure 1). The atoll was declared an IUCN Strict Nature Reserve in 1981, a UNESCO World Heritage Site in 1982 and a Ramsar Site in 2010 (Ramsar Convention on Wetlands 2010; UNESCO World Heritage 2023). Since 1979, ecological monitoring and scientific research on the atoll have been managed by the Seychelles Islands Foundation (SIF), which maintains a permanent research station on Picard.

Aldabra's climate is influenced by the prevailing wind direction, with the wet northwest monsoon bringing 75% of the yearly rainfall (mean 975 mm year^−1^; Haverkamp et al. 2017) and calmer weather from November to April, and the drier southeast trade winds bringing stronger winds from May to October. Seasonal changes drive variation in food and water availability and the amount of shade offered by foliage, which affects tortoise movement and activity (Gibson and Hamilton 1983). In particular, tortoise availability along transects may vary with access to freshwater, shade, thermal refugia and local aggregation around ephemeral pools or forage patches. Although Aldabra giant tortoises are generalist feeders, we are not aware of evidence for strong, predictable seasonal shifts towards particular feeding habitats that would consistently alter wet‐season availability along transects.

### 2.2 Data Collection

In 2017 SIF updated its monitoring protocols to include distance sampling—recording the perpendicular distance of individual tortoises from a central line transect. Tortoise sighting data were collected on Aldabra over 5 years (2018–2022 inclusive) using an adapted conventional distance sampling method (Buckland et al. 2001). Eighteen transects of varying length were established across the atoll (Figure 1) and divided into 50 m analytical sections with habitat type recorded per section (Table 1). Where a change in bearing was unavoidable (e.g., access/safety) the next 50 m section was laid out with a new centreline and distances were measured perpendicular to that active section to adhere to the assumptions of distance sampling (Thomas et al. 2010). Throughout the manuscript, we use ‘section’ to refer to each 50 m analytical unit. In the accompanying model code, ‘site’ is used for the same 50 m transect sections. Transects were distributed across the four main islands: nine on Grande Terre, five on Picard, three on Malabar and one on Polymnie. There are currently no tortoises on Polymnie so this transect was excluded from our analysis. Two observers walked each transect once per season between sunrise (06.00–07.00 h) and 09:00 h (tortoises tend to seek shade in the hotter temperatures after 09.00 h), survey timing within the season varied among years depending on logistics, staffing and access. Legacy transects refer to established monitoring lines that were purposively placed to proportionally span habitats found on Aldabra (Table 1); while not randomly allocated, our hierarchical model treats these as representative samples of the broader habitat strata to generate atoll‐wide estimates.

**TABLE 1:** Habitats on Aldabra included in giant tortoise monitoring (with descriptions and total habitat area across the atoll from Walton et al. 2019).

Habitats	Code	Description	Total habitat area (km^2^)	N of 50‐m transect sections
Mangroves	MAN	Homogeneous area of several mangrove tree species.	17.2	10
Open mixed scrub	OMX	Woody plants at low density. Ground layer vegetation has a high percentage cover of tortoise turf. Dicotyledonous herbs and long grasses are rare.	25.4	149
Standard mixed scrub	SMX	Similar to open mixed scrub but woody plants at a higher density. Dominated by *Fimbristylis cymosa* and *Cyperus niveus*. Tortoise turf is rare or absent.	42.3	100
*Pemphis* scrub	PEM	Homogeneous habitat dominated by *Pemphis acidula* .	35.8	68
Grassland	GRA	Homogeneous areas of tortoise turf grass.	4.5	33
Exposed surface	EXP	Limited vegetation cover, absent in some areas and, where present, dominated by tortoise turf. Bare substrate composed of champignon (deeply pitted limestone), sand or platin.	20.1	53
Champignon	CHP	Dominated by areas of champignon rock, with minimal vegetation.	4.8	13
Sand	SND	Dominated by sand substrate and some ground‐level vegetation in some areas. Includes dried‐out seasonal pools on Grande Terre.	2.8	4

Environmental conditions and staffing limitations affected sampling effort over the study period (Table 2), resulting in some transects not being sampled every year. Consequently, we modelled population counts on a section‐ and year‐specific basis. Field observations and prior work indicate that during the dry season tortoises more often use dense cover and thermal refugia, which reduces availability for detection, making underestimated counts and population estimates more likely. For this reason, we focus our analysis on the wet season counts (dry season analysis can be found in the Appendix— – Figures S1–S3). For the purposes of this study each sampling ‘year’ corresponds to the wet season beginning in November of the previous calendar year; for example, data collected from November 2022 through April 2023 are reported as part of the 2022 wet season. The dry season spans May to October and is attributed to the same calendar year.

**TABLE 2:** Sampling effort for giant tortoise population monitoring across Aldabra during the wet seasons (Nov–Apr) of 2018–2022. Total area sampled assumes a 50 m truncation distance either side of the transect line. The year column indicates the year in which the wet season sampling period started, that is, for 2018, the sampling period started in November 2018 and finished in April 2019, with each of the 425 transect sections recorded walked once during this period.

Wet season	Total # of sampled transect sections	Total area sampled km^2^ (% of total atoll area)
2018	425	2.13 (1.37)
2019	430	2.15 (1.38)
2020	417	2.09 (1.34)
2021	430	2.15 (1.38)
2022	227	1.14 (0.73)

### 2.3 Statistical Analysis

The inaccuracy of estimates of abundance is often due to two main factors: failure to sample the full range of habitats and conditions and counts biassed by imperfect detection of individuals (Buckland et al. 2008; Yoccoz et al. 2001). Although Aldabra has a long history of organised monitoring, challenges remain due to uneven sampling effort across different habitats and vegetation types, and the inherently imperfect detection probabilities of giant tortoises in dense or complex terrain. Thoughtful study design (Greenwood and Robinson 2006) can address habitat and sampling gaps while imperfect detection can be mitigated using capture–mark–recapture (Borchers et al. 2002; Royle and Dorazio 2008) or distance sampling (Buckland et al. 2001) methods. These methods adjust raw counts with estimated detection probabilities to produce true abundance estimates (Kery et al. 2005; Kery and Royle 2010).

To directly address the specific issues identified in the Aldabra monitoring data—particularly uneven sampling effort with regards to both total sampling area and representation of habitat types in those samples (Table 2), imperfect detection across varying habitats and sparse data at certain sections—we developed a Bayesian hierarchical distance sampling model using data augmentation (Kéry and Royle 2016; Link and Barker 2010; Royle and Dorazio 2008). The model has two distinct components: (1) the observation process, which describes how individuals are observed, and (2) the abundance (or process) model, which describes how individuals are distributed (Kéry and Royle 2016).

### 2.4 Model Definition

Here we provide a summary of the model structure aimed at non‐experts, followed by a full model definition describing hierarchical structure, likelihoods and priors.

We estimated tortoise abundance using a hierarchical Bayesian state‐space model based on the framework described by Royle et al. (2004). This approach separates two key processes: the observation process which describes how tortoises are detected and the ecological process which describes how many tortoises are actually present.

It is standard in line transect distance‐sampling to dismiss observations lying beyond a certain distance as they are rarely detected reliably. In our analysis tortoise observations were right‐truncated at 50 m. The remaining observations are known to be less likely with increasing distance from the transect—to account for this the model estimates a detection function which describes how the detection probability decreases with increasing distance and allows counts observed further from the transect to be adjusted accordingly. There is a basic assumption that all tortoises present on the transect line are seen with probability 1: this seems reasonable given the huge size of the target organism and is supported by visual inspection of detection data. We compared potential detection functions on the pooled (5 years) data using the ‘Distance’ package (Miller et al. 2019). Although the hazard‐rate model was preferred based on Akaike Information Criterion comparisons, this function led to unstable and unreliable abundance estimates when fitted within our model (see Appendix S6 for comparison). We therefore modelled the observation process using the more robust half‐normal function and checked model fit visually by habitat (see Appendix S7).

Not all individuals present during a survey are recorded and this must also be accounted for by the model. To do this we employed data augmentation (Royle et al. 2007; Tanner and Wong 1987), whereby the model assumes that the observed individuals are part of a larger ‘unknown’ population that may exist. The model then estimates how many of these individuals are likely to be real given the detection process.

Variation in tortoise abundance across sections and years was modelled using a hierarchical structure that allows information to be shared across locations. This approach helps to stabilise the estimates at sections or in habitats where data are sparse by partially pooling the section‐level estimates towards an overall mean.

#### 2.4.1 Observation Model

For efficient computation when the log‐likelihood lacks a standard form, we apply the zeros trick (Lunn et al. 2000) which implements the custom likelihood for the distance observations. To do this we use a set of dummy observations $y_{i}$ set to 0 and model their probability under a Poisson distribution:

(1) $$y_{i}\sim \text{Poisson}\left(\lambda_{i}\right)$$

So that $\log P\left(y_{i}=0|\lambda_{i}\right)=-\lambda_{i}$. Here $\lambda_{i}$ is the negative log‐likelihood contribution of observation i at distance $d_{i}$ calculated as:

(2) $$\lambda_{i}=-\left(\frac{-{d_{i}}^{2}}{2{\sigma_{s,o}}^{2}}-\log\left({\bar{p}}_{,s,o}\right)-\log\left(B\right)\right)+C$$

where *C* is a large constant ensuring $\lambda_{i}>0$ and B is the truncation distance (50 m). $\sigma_{s,o}$ is the scale parameter of the detection function for section s and sampling occasion o. ${\bar{p}}_{s,o}$ represents the integrated detection probability across the truncation distance:

(3) p¯s,o=σs,oπ22ΦBσs,o−1B

where Φ represents the cumulative distribution function of the standard normal distribution. This functional form provided a robust fit to the empirical distance data across all habitat types (see Figure S7 for observed distance histograms with overlaid detection functions). The effects of habitat type on detection are incorporated through a log‐linear relationship:

(4) $$\log\left(\sigma_{s,o}\right)=\beta_{v}$$

where $\beta_{v}$ is the coefficient associated with habitat type v, assumed consistent across years and transect sections.

#### 2.4.2 Abundance Model

The abundance process is modelled using section‐ and occasion‐specific effects $\gamma_{s,o}$, where each ‘occasion’ represents a unique section‐year combination. These effects determine the logit‐scale availability $\psi_{s,o}$ of individuals (the data augmentation parameter):

(5) $$\text{logit}\left(\psi_{s,o}\right)=\gamma_{s,o}$$

To ensure parameter identifiability $\gamma_{s,o}$ follows a hierarchical structure with a shared variance component (see Figures S4 and S5):

(6) $$\gamma_{s,o}\sim \text{Normal}\left(\mu_{\gamma ,s},\sigma_{\gamma }\right)$$

In this formulation, $\mu_{\gamma ,s}$ represents the long‐term mean availability for section s (on the logit scale) while $\sigma_{\gamma }$ is a global standard deviation representing the magnitude of year‐to‐year fluctuations shared across all sections. This hierarchical approach encourages ‘shrinkage’ towards a shared distribution which helps mitigate estimation uncertainty in sections with limited data. The hierarchical shrinkage prior ensures that section‐specific estimates are informed by the overall distribution, reducing overfitting in cases where individual sections have sparse observations.

#### 2.4.3 Linking Observation and Abundance Processes

We link the observation and abundance processes through joint likelihoods. The number of detections at a given section and sampling occasion $n_{s,o}$ follows a binomial distribution:

(7) $$n_{s,o}\sim \text{Binomial}\left({\bar{p}}_{s,o},N_{s,o}\right)$$

where $N_{s,o}$ represents the true number of individuals in section s on occasion o, following:

(8) $$N_{s,o}\sim \text{Binomial}\left(\psi_{s,o},M\right)$$

where M is the total number of possible individuals (both observed and augmented), and $\psi_{s,o}$ represents the probability that an individual is real.

We fit the described model using Markov chain Monte Carlo (MCMC) sampling using the NIMBLE package (de Valpine et al. 2017) in R version 4.3.0 (R Core Team 2022). We ran three independent MCMC chains for 250,000 iterations and burn‐in of 75,000. We assessed convergence through a visual inspection of the chains and by using the Gelman‐Rubin diagnostic (Brooks and Gelman 1998). We assumed convergence had been reached when chains were substantially overlapping, and the Gelman‐Rubin diagnostic was < 1.1 for all monitored parameters (see Table S1 for a summary of convergence diagnostics). We assessed model fit by generating posterior predictive plots that displayed the expected counts and their 95% credible intervals for each section and occasion combination, with the actual data overlaid (see Appendix). The model predictions successfully included the actual counts for all the sampled sections/occasions. All code and data are available (see Appendix for details).

### 2.5 Prior Distributions

We assigned weakly informative priors to all model parameters. Detection coefficients (β) and section‐level mean abundances ($\mu_{\gamma ,s}$) followed a Normal (0, SD = 10) distribution. For the abundance process, we implemented a hierarchical shrinkage prior where each section‐occasion effect ($\gamma_{s,o}$) was drawn from a Normal distribution centred on the section‐level mean ($\mu_{\gamma ,s}$). The magnitude of year‐to‐year fluctuations across sections was governed by a global standard deviation ($\sigma_{\gamma }$) assigned an Exponential distribution (rate = 1) prior to constrain variability within a biologically reasonable range while allowing the data to drive the posterior estimates.

### 2.6 Population Estimates

It is important to clarify the demographic scope of the population estimates presented here. Juvenile tortoises exhibit cryptic behaviour, often seeking refuge in dense vegetation, which makes them effectively unavailable for detection. Consequently, the estimates of abundance and the resulting densities should be interpreted as applying primarily to the adult and sub‐adult components of the population. We did not apply a fixed size or mass threshold because these are inconsistent across the different islands; ‘sub‐adult’ is used operationally to describe visible non‐juvenile individuals. To generate atoll‐wide estimates and ensure the full propagation of uncertainty we performed the extrapolation on each iteration of the MCMC posterior. For each sampling year we aggregated the posterior abundance $\left(N_{s,o}\right)$ across all sampled sections within each habitat type. The density for each habitat was calculated by dividing the total abundance by the total area of the sampled strips $\left(2\times \text{total line length}\times \text{truncation distance}\right)$. We then extrapolated these densities to the entire atoll (excluding Polymnie) by multiplying by the mapped habitat areas from Walton et al. (2019). This procedure is equivalent to direct scaling by habitat area (Miller et al. 2004) but makes the detectability correction explicit via the fitted detection function.

### 2.7 Alternative Approaches

To evaluate the robustness of our modelling approach, we conducted a sensitivity analysis using other commonly applied distance‐sampling frameworks. We fitted (i) a stratified conventional distance‐sampling model using the *Distance* package and (ii) several integrated hierarchical distance‐sampling models using the gdistsamp function in the *unmarked* package. These approaches produced broadly comparable point estimates in well‐sampled years but proved unstable when applied to the full Aldabra dataset. Models struggled to accommodate the uneven sampling across habitats and years, which led to convergence failures in complex models aimed to mimic the structure of the Bayesian model. With more parsimonious models, the parameters produced biologically unrealistic temporal variation in population estimates. A full description of these alternative analyses is provided in the Appendix and see Figure S8.

## 3 Results

**FIGURE 2:**
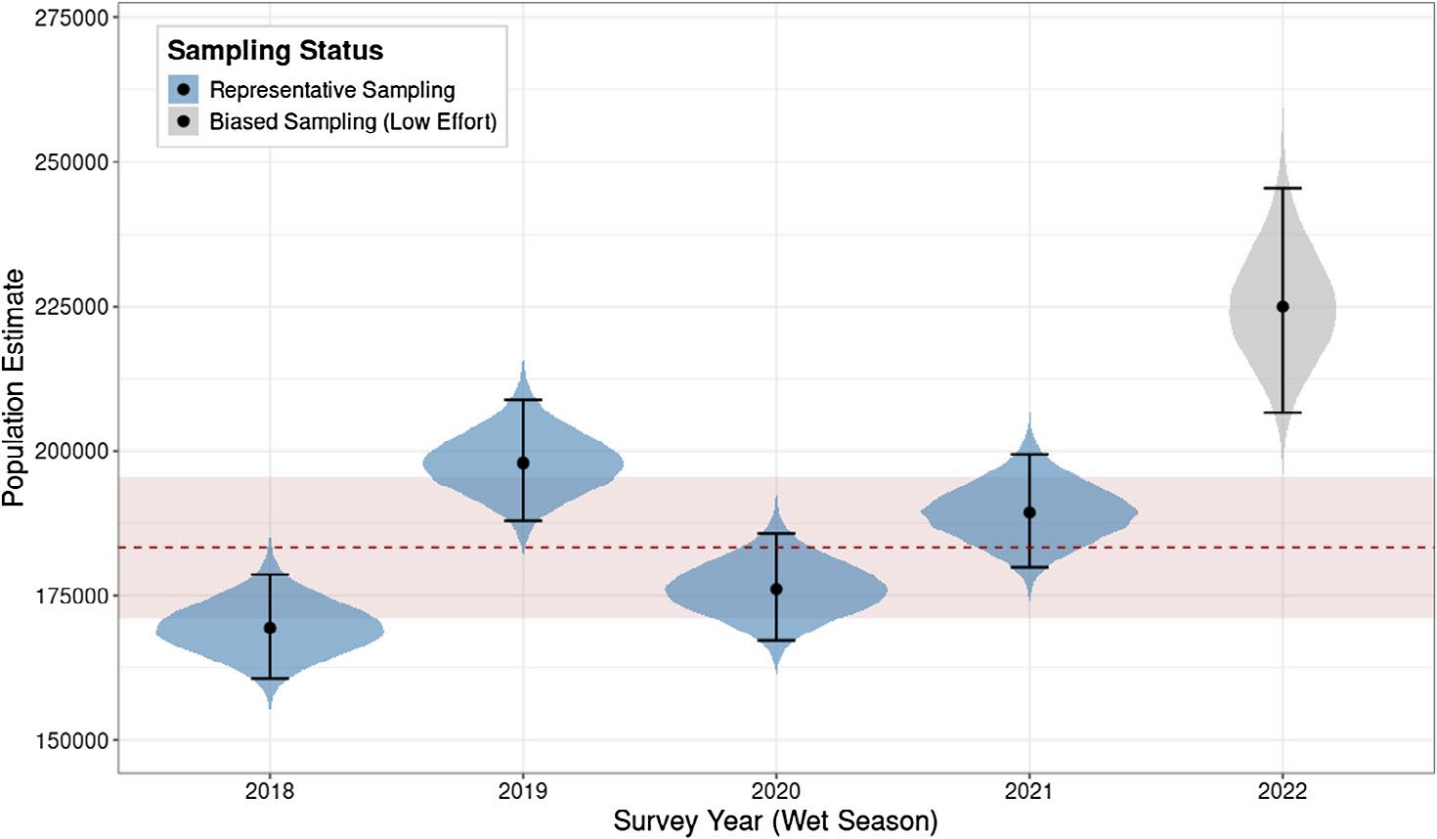
Violin plots of posterior population estimates for the Aldabra giant tortoise on Aldabra, excluding Polymnie (2018–2022 inclusive). Dashed red line indicates overall mean with shaded red area showing ±1 SD; 2022 estimate (in grey) has been excluded from calculations due to sampling effort being low and failing to meet the required threshold area. Sections covered in 2022 were also biassed towards high‐density areas—see 4. Discussion.

Model fit was very good, with all observed counts falling within the 95% credible intervals of the corresponding model‐predicted values across all section and year combinations. Tortoise population estimates were relatively stable across the sampling period 2018–2021, with a mean population estimate of 183,274 (SD = 12,172; Figure 2). Sampling effort in the wet season that ran from November 2022 to April 2023 (recorded as 2022 in Figure 2) was considerably lower than previous years and the associated population estimate is higher than the preceding years; therefore, this has been excluded from the mean estimate due to an inherent bias in sampling (see Discussion for further explanation).

**FIGURE 3:**
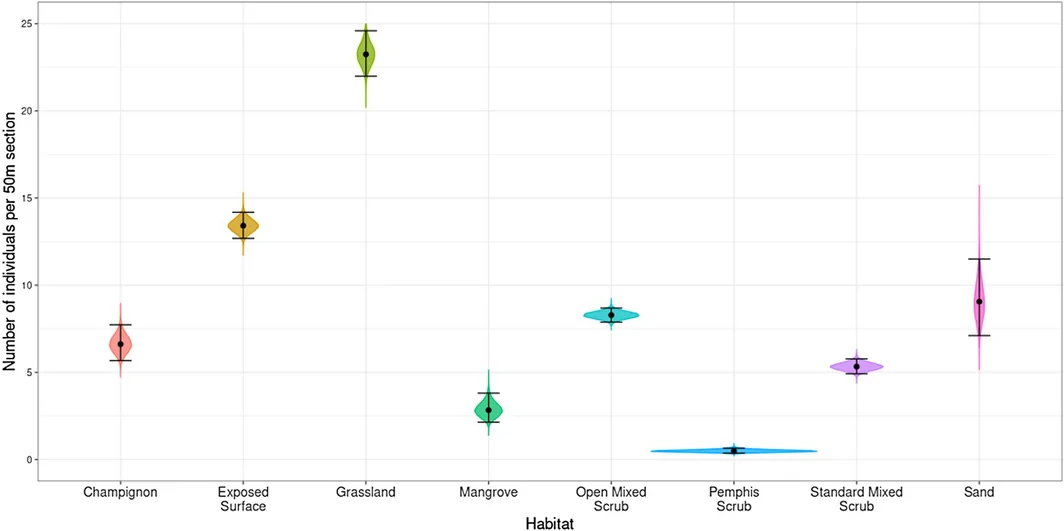
Detection‐corrected posterior estimates of average tortoise numbers per 50 m transect section by habitat (wet season). Violins represent the posterior distribution of the mean latent abundance per transect section ($N_{s,o}$; state scale) calculated across all MCMC iterations to ensure full propagation of uncertainty. Dots are posterior medians and bars are 95% credible intervals. These estimates account for imperfect detection and vary by habitat‐specific detectability $\left(\sigma_{\mathrm{veg}}\right)$.

To compare abundance among habitats we used output from the HDS model to estimate detection‐corrected density (number of individuals per 50 m transect section) for each habitat (Figure 3). For each posterior draw we summed the estimated number of individuals across all section–year combinations within a habitat and divided this total by the corresponding surveyed area which produces a posterior distribution of density for each habitat type. Wider or taller violins indicate greater uncertainty—typically arising from habitats with less sampled area or fewer detections. Figure 3 indicates that grassland habitats supported the highest densities of tortoises, followed by exposed surfaces and open mixed scrub. Sand habitats also appear to contain relatively high densities, although this category includes only four transect sections and therefore exhibits greater uncertainty.

**FIGURE 4:**
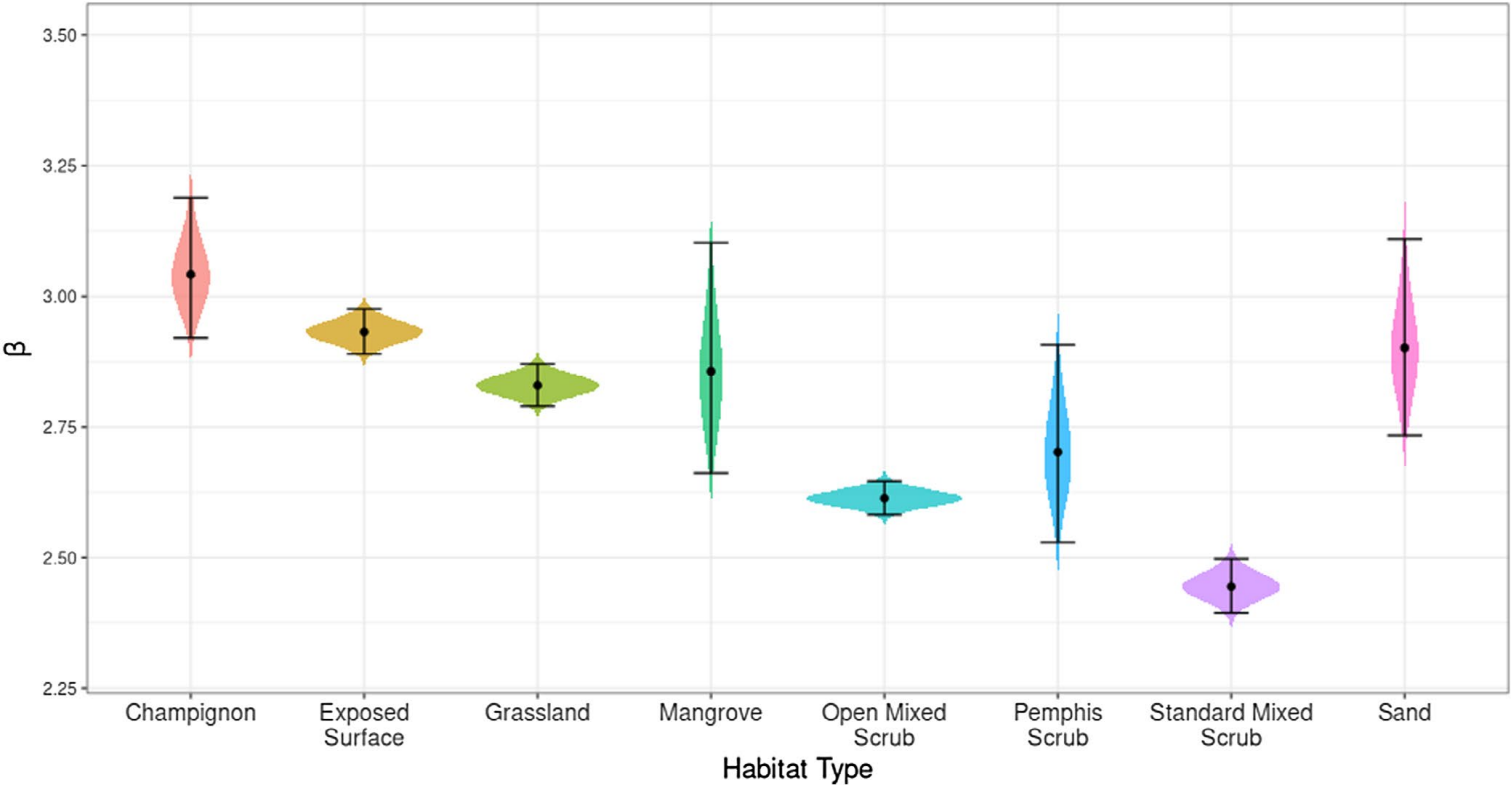
Posterior distributions of the habitat‐specific *β* coefficients, shown as violin plots. In the detection model, *β* parameterises the half‐normal scale as $\log\left(\sigma \right)=\beta_{v}$, where σ is the scale parameter controlling how quickly detection probability declines with distance from the transect. Higher *β* (and thus higher σ) indicates a broader detection function and therefore greater detectability at a given distance.

The habitat effects on the observation process are described by the detection coefficients (*β*) which determine the scale parameter of the detection function (Figure 4). Larger *β* values correspond to a larger detection scale parameter (*σ*) which results in flatter detection functions where individuals remain detectable at greater distances from the transect line. Tortoises were generally detectable at greater distances in more open habitats and less detectable in denser scrub habitats. Uncertainty was greatest in mangrove and Pemphis‐scrub habitats reflecting both lower sampling representation and relatively small numbers of observed tortoises in these environments.

## 4 Discussion

### 4.1 Tortoise Population Estimate and Habitat Preference

We estimate Aldabra's tortoise population to be 182,424 (SD = 13,257) individuals and apparently relatively stable between 2018 and 2021, reinforcing our understanding of space use and behaviour. Our estimate is higher than all previously published estimates (33,000—Gaymer 1973; 150,466 [134,020–166,907 95% CI] Bourn and Coe 1978) including the most recent estimate of 100,000 individuals in 1999 (Bourn et al. 1999). These earlier estimates relied on designs and analyses that did not explicitly model detectability or consider seasonal availability, thus making strict comparisons difficult. Our findings suggest that these previous estimates likely provided underestimates of abundance by failing to account for tortoises that were present but undetected at greater distances from the observer. The Gaymer (1973) study was primarily a marking methods exercise rather than a robust atoll‐wide census; Bourn and Coe (1978) scaled raw counts from ~1 ha plots without detection modelling and the 1997 resurvey used different season/timing and lower sampling intensity in key areas with transformed counts, ad hoc adjustments but still no detection model (Bourn et al. 1999). Subsequent transect analyses demonstrated persistence/trends but were based on purposively placed lines and did not yield a detection‐corrected atoll‐wide estimate of abundance (Turnbull et al. 2015). Our analysis utilises a Hierarchical Distance Sampling (HDS) framework (Royle et al. 2004) which separates the observation process (detection) from the underlying ecological process governing abundance. By explicitly modelling detection and abundance separately we robustly estimate the number of individuals and provide a comparable, detection‐corrected baseline for future monitoring. Our estimates refute the previously held notion that the population was at carrying capacity in the late 1900s (Gerlach et al. 2013) and instead suggest that carrying capacity on Aldabra may be a dynamic limit that has shifted following the recovery of vegetation and removal of invasive predators. Our results support previous long‐term monitoring data analysis which showed no sign of significant decline (Turnbull et al. 2015) and suggest a possible population increase since earlier estimates. Although the most recent estimate from 2022 might suggest a continuing population increase, sampling effort was considerably lower that year and the transects surveyed in the 2022 wet season (see Appendix) were those with historically greater tortoise counts—probably causing a biassed‐upwards population estimate. More data are required to confirm any potential trends moving forwards, but this information will help to inform best practice to improve sampling protocols in the future (see Section 4.2).

Our findings reinforce previous understanding of tortoise habitat preference (Swingland and Frazier 1980; Turnbull et al. 2015; Walton et al. 2019)—the detection‐corrected estimates (Figure 4) indicate that tortoises are likely to occur in their highest number in ‘tortoise turf’ grassland with exposed surface and open mixed‐scrub habitats also showing elevated numbers. We emphasise that these are assumptions, not demonstrated preferences or selection. Some habitat types are poorly represented in the dataset due to their low total area on Aldabra (e.g., sand, with only 4 transect sections sampled each year) meaning these estimates should be interpreted with some caution. Habitat enters the detection function (log‐*σ*) meaning these contrasts reflect differences in abundance rather than artefacts of detectability. For transparency we also plot section‐level γ (ψ) summaries from the data augmentation component in the Appendix (Figures S4 and S5); we do not use these as evidence for habitat effects but note that they are largely concordant with the density‐based ranking.

Aldabra giant tortoises are active throughout the day but typically seek shade to avoid overheating during the hotter parts of the day (Falcón et al. 2018). Our surveys were conducted before 09:00 h so the habitat patterns we report reflect night/morning space use rather than full‐day activity. This timing may partly explain the higher pooled densities in more open habitats (exposed surface and sand) where early‐morning movement is possible. By contrast, mixed‐scrub habitats plausibly support substantial densities because they provide shade later in the day. Relatively shadeless coastal substrates (e.g., exposed limestone and sand) and intertidal mangroves show lower densities, consistent with previous observations (Swingland and Frazier 1980; Walton et al. 2019). Uncertainty is greater in sparsely sampled or low‐encounter habitats which is reflected in wider credible intervals.

We expected to uncover seasonal habitat associations in line with historical research (Bourn and Coe 1978; Gibson and Hamilton 1983) but found little evidence to support this with broadly similar count proportions across both seasons (although reduced numbers in the dry season) except for a slightly elevated estimate for the number of individuals associated with mixed scrub habitats in the dry season (dry season analysis in the Appendix S1–S3). Our results are more in line with a more recent GPS tracking analysis of the tortoises (Walton et al. 2019) that found no evidence for seasonal habitat preference. Section‐mean variation was higher across all habitat types in the dry season, suggesting that tortoises exhibit more flexible habitat use during this period when there is less shade and water availability.

### 4.2 Seasonal Variation and Sampling Recommendations

We chose to focus our analysis and inference on wet season counts as this is when the greatest number of tortoises are available for detection. The atoll is geographically isolated so individuals cannot leave the population and their life history is sufficiently slow that seasonal population variation is unlikely to be caused by fluctuations in birth/death processes. Aldabra's tropical climate is also not consistent with true brumation. Instead, reduced counts and lower posterior estimates during the dry season suggest a form of ‘temporary emigration’ where individuals are unavailable for detection rather than simply being missed by observers. This distinction is important as it implies availability bias rather than a failure of the detection function. By focusing our primary analysis on the wet season we satisfy the assumption of population closure and ensure the majority of the population is available for detection in the transect centreline. While alternative distance sampling models that account for temporary emigration have been proposed (e.g., Chandler et al. 2011; Sollmann et al. 2015), their performance is better suited to repeated sampling of defined transect sections consistently through time—for exmple, over multiple days within a season (Kéry and Royle 2016). We were unable to achieve satisfactory mixing of the MCMC chains likely due to the irregular nature and temporal coverage of our data. Aldabra giant tortoises have been found to change their movements and foraging behaviour depending on the season and resource availability (Gibson and Hamilton 1983; Walton et al. 2019), and our analysis (see Appendix) indicates that more tortoises are available for detection in the wet season. Accordingly, prioritising wet‐season surveys—when availability to detection is highest—should yield more precise and representative population estimates than dry‐season counts.

Recent changes in climate, notably increased droughts on Aldabra (Haverkamp et al. 2017), may be responsible for the somewhat unanticipated seasonal variation in availability for detection. Indeed, abrupt changes to precipitation patterns across East Africa, including the failure of prolonged rains, have been linked to changes in sea surface temperatures (Lyon and DeWitt 2012). Aldabra's significant increase in the number of drought months per year (Haverkamp et al. 2017) could have marked impacts on tortoise behaviour and population trend, as longer drought periods reduce vegetation recovery and giant tortoises are unlikely to be able to respond rapidly to such selection pressures due to their slow life history (Falcón and Hansen 2018; Haverkamp et al. 2017). As such, tortoise behaviour and distribution in different seasons may become less predictable than previous studies have suggested (Gibson and Hamilton 1983). Based on these evaluations, differences between seasons are likely to be increasing in the changing environment (Falcón and Hansen 2018).

Tortoises are also thought to congregate around ephemeral pools during even short periods of low rainfall, which are often far from sampling transects on the island (Falcón and Hansen 2018; Merton et al. 1976). Whilst dry season sampling is currently less valuable for population estimations, it still provides an opportunity to investigate seasonality and, as the dataset grows, would allow inference on temporary emigration parameters. Dry season sampling is therefore recommended every 2–4 years.

### 4.3 Evaluation of Methods and Wider Implications

Our results emphasise the importance of maintaining a large, representative sample across years and habitat types for accurate analysis and year‐to‐year comparisons. Although we recognise that our approach is perhaps more complex than many environmental NGOs would typically conduct in‐house, it does provide great benefit in understanding limitations, barriers and potential efficiencies when estimating population sizes of target species. Irregular monitoring is commonplace in logistically challenging and remote sections, especially for many conservation and environmental NGOs with high staff turnover and seasonality. Whilst we acknowledge the benefit of a standardised monitoring protocol that would be more easily analysed by standard analytical techniques, these are frequently unfeasible in real world situations. Our comparisons with alternative established distance sampling analyses (see Appendix) confirm they can struggle with sparse data and often fail to propagate the full uncertainty of the detection process. Our choice of inference model delivers an informed estimate even in the absence of regular sampling data.

Roughly half as many 50‐m transect sections were sampled in 2022 when compared to previous years and, whilst looking at historic tortoise distribution, we found that the sections missed were typically those of lower tortoise abundance. Consequently, a larger proportion of the atoll total relied on extrapolation, yielding higher estimates than previous years (Miller et al. 2004). While the 2022 estimate should be interpreted cautiously, we have included it to demonstrate the robustness of our model (as the posterior predictive plot reveals why this pattern emerges) and to add weight to our recommendations to modify sampling protocols. The 2022 estimate highlights the need for sufficient data to ensure reliable analysis (Miller et al. 2004; Thomas et al. 2010). Projects using distance sampling, such as the one conducted by SIF, would benefit from establishing a minimum sampling area threshold to ensure adequate data collection for more accurate total population estimates (Miller et al. 2004).

When looking at the distribution of 50‐m transect sections across habitats, some habitat types were poorly represented. Although these proportions loosely represent the proportions of habitat cover across the atoll, they provided limited information for associated population estimates, leading to greater variation and uncertainty. Sampling low‐density habitats introduces a trade‐off: while increasing transect sections in these habitats would improve the certainty of their estimates, it would require substantially more effort for minimal gain given the near‐zero tortoise counts typically observed there. Consequently, researchers must decide whether to include or exclude such habitats based on study objectives. Where inclusion is necessary, we suggest a practical minimum of 15–20 transect sections per habitat type to ensure sufficient data for reliable estimates and reduce uncertainty.

Monitoring could be repeated yearly or every other year, but crucially, monitoring effort should meet the minimum sampling threshold of 1% land coverage (Goldsmith and Sutherland 1997). In the case of Aldabra this would be 1.55 km^2^, and this is being met by the current monitoring design which covers 2.15 km^2^. Given their long lifespans and slow maturity, monitoring giant tortoises should include individual‐based approaches and targeted studies of hatchling and juvenile survival. This combined strategy will provide more timely demographic insights than monitoring adults alone, helping to identify thresholds for concern and trigger appropriate management actions earlier.

The results also highlight the remarkable conservation success of this previously exploited population, attributed to long‐term protection and restoration efforts including the eradication of invasive species (Bunbury et al. 2018, 2019). Despite the ongoing threats of invasive mammal presence on Aldabra and of increased drought periods (Haverkamp et al. 2017), the giant tortoise population remains apparently relatively stable. Eradication of invasive mammals, which is planned, would reduce tortoise egg and hatchling predation, potentially removing barriers to further growth of the tortoise population (Spatz et al. 2022) and potentially allowing the tortoise population to better adapt to future changes in climate. More widely, restoration programmes using translocated tortoises in rewilded ecosystems should focus on ensuring that their tortoise populations are robust enough to withstand and adapt to climate changes by addressing threats such as invasive alien mammals and poaching. Given the native Aldabra giant tortoise population's increase and current stability following historically low numbers, rewilding projects using Aldabra tortoises remain a viable conservation strategy.

We used a Bayesian approach to clarify the hierarchical structure of distance sampling by involving two models: the process model (abundance/distribution of individuals) and the observation model (detection/non‐detection of individuals) (Kéry and Royle 2016) We were able to model habitat variation whilst characterising the uncertainty associated with any estimates (Clark et al. 2005; Cressie et al. 2009). By implementing a partial pooling approach with a global variance component $\left(\sigma_{\gamma }\right)$, we ensured model identifiability and stability across diverse habitat types. This hierarchical framework effectively ‘borrows strength’ from data‐rich areas to inform estimates in data‐sparse strata, a significant advantage over non‐hierarchical methods that often fail to converge under irregular sampling designs. We effectively accounted for individuals that may not have been detected by utilising data augmentation, thus improving the robustness of our abundance estimates even with incomplete or sparse data (Cressie et al. 2009). Additionally, by incorporating a shrinkage prior, we stabilised estimates in data‐sparse regions and ensured that abundance predictions remained reliable despite variability in detection effort.

This approach allowed for more flexible and accurate modelling of population dynamics, providing information on tortoise behaviour, movement, and detection to help inform management and future monitoring of Aldabra giant tortoises. We encourage the use of this method in generating future estimates of both the original giant tortoise population on Aldabra Atoll and translocated populations in rewilded ecosystems. More widely, this method would also benefit long‐term terrestrial population monitoring with unmarked individuals, particularly in species that inhabit diverse habitat types.

## Author Contributions

**Lara B. Hillyard:** data curation (equal), formal analysis (equal), investigation (equal), methodology (equal), writing – original draft (equal), writing – review and editing (equal). **Nancy Bunbury:** conceptualization (equal), methodology (equal), project administration (equal), supervision (equal), writing – review and editing (equal). **Christopher N. Kaiser‐Bunbury:** conceptualization (equal), supervision (equal), writing – review and editing (equal). **Dave Hodgson:** methodology (equal), supervision (equal), writing – review and editing (equal). **Luke A'Bear:** data curation (equal), investigation (equal). **Cheryl L. Sanchez:** data curation (equal), investigation (equal), writing – review and editing (equal). **Christopher W. Jones:** data curation (equal), investigation (equal), writing – review and editing (equal). **April Burt:** data curation (equal), writing – review and editing (equal). **Frauke Fleischer‐Dogley:** conceptualization (equal), methodology (equal), project administration (equal), writing – review and editing (equal). **Dave W. Hudson:** formal analysis (equal), methodology (equal), project administration (equal), supervision (equal), writing – review and editing (equal).

## Funding

This work was supported by Seychelles Islands Foundation and the University of Exeter. This research did not receive any specific grant from funding agencies in the public, commercial, or not‐for‐profit sectors.

## Conflicts of Interest

The authors declare no conflicts of interest.

## Supporting information


**Figure S1:** Violin plots of posterior predicted dry season population estimate for the Aldabra giant tortoise on Aldabra (2018–2022 inclusive). Solid red line indicates overall mean, dashed lines show ±1 SD; 2022 estimate (in grey) has been excluded from calculations due to sampling effort being only 50% of other years and sections were high‐density areas.
**Figure S2:** Posterior distributions of *β* coefficients for each habitat type, shown as violin plots. These *β* values determine the log‐scale detection distance (*σ*) in each habitat, with higher values indicating a greater effective detection distance. Higher values reflect habitats where individual tortoises are more easily detected.
**Figure S3:** Detection‐corrected posterior estimates of average tortoise numbers per 50 m transect section by habitat (dry season). Violins show the posterior distribution of the latent abundance per transect section ($N_{s}$; state scale) from the Bayesian hierarchical distance‐sampling model with data augmentation; dots are posterior medians and bars are 95% credible intervals. For each draw, section‐level $N_{s}$ values were averaged within habitat to give a mean individuals per 50 m transect section.
**Figure S4:** (Top) Posterior distributions of section‐specific mean occupancy $\left(\mu_{\gamma ,s}\right)$, grouped by habitat type (wet season); (Bottom) Posterior distribution of the global standard deviation ($\sigma_{\gamma }$) for section‐level fluctuations (wet season).
**Figure S5:** (Top) Posterior distributions of section‐specific mean occupancy $\left(\mu_{\gamma ,s}\right)$, grouped by habitat type (dry season); (Bottom) Posterior distribution of the global standard deviation ($\sigma_{\gamma }$) for section‐level fluctuations (dry season).
**Figure S6:** Plots of Hazard‐rate (top) and Half‐normal (bottom) detection functions from fitted models in Distance fitted to pdf of observed distance data truncated at 50 m.
**Figure S7:** Comparison of observed distance histograms and fitted habitat‐specific detection functions. Grey bars represent the rescaled density of observed tortoise distances from the transect centreline, aggregated into 10 m bins to smooth localised sampling noise and ‘heaping’ (rounding) common in field‐collected distance data. The coloured lines represent the posterior mean half‐normal detection function for each of the eight habitat types.
**Table S1:** Summary of MCMC convergence diagnostics for the HDS model (wet season). Diagnostics are summarised for primary parameter groups across three independent chains (250,000 iterations per chain). $\hat{R}$ represents the Potential Scale Reduction Factor (Gelman‐Rubin diagnostic), which compares the within‐chain and between‐chain variance; values < 1.1 are widely accepted as evidence of chain convergence (Brooks and Gelman 1998). The Effective Sample Size (ESS) represents the number of independent, non‐autocorrelated samples generated by the MCMC algorithm. For each parameter group, we report the median value and the ‘worst‐case’ diagnostic (Maximum $\;\hat{R}$ and Minimum ESS) to demonstrate the robustness of the posterior estimates across all 430 sampling locations and 8 habitat strata.
**Figure S8:** Comparison of atoll‐wide population trajectories across five modelling frameworks. Shaded ribbons represent 95% Credible Intervals for the Bayesian model and 95% Confidence Intervals for frequentist models. For models 1, 2.2, 2.3, 2.4 and 2.5 uncertainty was propagated into the atoll‐wide total using a parametric bootstrap (500 iterations), where coefficients were sampled from the multivariate normal distribution defined by the model's maximum likelihood estimates and variance–covariance matrix.

## Data Availability

Data and main analysis code are stored in a Dryad repository with DOI https://doi.org/10.5061/dryad.0p2ngf2hj or alternatively accessible via github here: https://github.com/davehudson67/TortoiseAnalysis.
