# Supplementary material for: Inferring the Abundance of an Island Megaherbivore From Semi‐Structured Distance Sampling
Source: Ecol Evol. 2026 Sep 4;16(9):e74282. doi: 10.1002/ece3.74282 (PMC13542991; doi:10.1002/ece3.74282)
Supplement: Supplementary file 1 [file ECE3-16-e74282-s001.docx]

# Appendix

- Dry Season Analysis (Figures S1-S3)
- Section level γ (ψ) summaries for both wet and dry season (Figures S4 & S5)
- Comparison plot of detection functions (wet season) (Figure S6)
- Fitted Detection Functions vs. Observed Distance Histograms (wet season) (Figure S7)
- Convergence Diagnostic Summary ($\hat{R}$) and Effective Sample Size (wet season) (Table S1)
- Sensitivity Analysis and Comparative Evaluation of Modelling Frameworks
- [Separate file – ‘traces.pdf’] Trace plots from the manuscript analysis
- [Separate file – ‘fits.pdf’] Posterior Predictive Check – model predicted counts vs actual counts from the manuscript analysis
- [Separate file – ‘preds.pdf’] Estimated population sizes across section-year combinations, summarising the true abundance from the manuscript analysis

1. **Dry Season Analysis:**

The following output matches the wet season output presented in the main manuscript but uses only the sampling counts from the dry seasons.

**Figure S1:** Violin plots of posterior predicted Dry season population estimate for the Aldabra giant tortoise on Aldabra (2018–2022 inclusive). Solid red line indicates overall mean, dashed lines show +/- 1SD; 2022 estimate (in grey) has been excluded from calculations due to sampling effort being only 50% of other years and sections were high-density areas

**Figure S2:** Posterior distributions of β coefficients for each habitat type, shown as violin plots. These β values determine the log-scale detection distance (σ) in each habitat, with higher values indicating a greater effective detection distance. Higher values reflect habitats where individual tortoises are more easily detected.

**Figure S3:** Detection-corrected posterior estimates of average tortoise numbers per 50m transect section by habitat (dry season). Violins show the posterior distribution of the latent abundance per transect section ($N_{s}$; state scale) from the Bayesian hierarchical distance-sampling model with data augmentation; dots are posterior medians and bars are 95% credible intervals. For each draw, section-level $N_{s}$values were averaged within habitat to give a mean individuals per 50 m transect section.

**Figure S4: (Top)** Posterior distributions of section-specific mean occupancy $\left( \mu_{\gamma,s} \right)$, grouped by habitat type (wet season); **(Bottom)** Posterior distribution of the global standard deviation ($\sigma_{\gamma}$) for section-level fluctuations (wet season).

**Figure S5: (Top)** Posterior distributions of section-specific mean occupancy $\left( \mu_{\gamma,s} \right)$, grouped by habitat type (dry season); **(Bottom)** Posterior distribution of the global standard deviation ($\sigma_{\gamma}$) for section-level fluctuations (dry season).

**Figure S6:** Plots of Hazard-rate (top) and Half-normal (bottom) detection functions from fitted models in Distance fitted to pdf of observed distance data truncated at 50m.

**Figure S7.** Comparison of observed distance histograms and fitted habitat-specific detection functions. Grey bars represent the rescaled density of observed tortoise distances from the transect centerline, aggregated into 10m bins to smooth localized sampling noise and "heaping" (rounding) common in field-collected distance data. The coloured lines represent the posterior mean half-normal detection function for each of the eight habitat types.

**Table S1. Summary of MCMC convergence diagnostics for the HDS model (wet season).** Diagnostics are summarized for primary parameter groups across three independent chains (250,000 iterations per chain). $\hat{R}$ represents the Potential Scale Reduction Factor (Gelman-Rubin diagnostic), which compares the within-chain and between-chain variance; values $\left< 1.1 \right.$ are widely accepted as evidence of chain convergence (Brooks & Gelman, 1998). The Effective Sample Size (ESS) represents the number of independent, non-autocorrelated samples generated by the MCMC algorithm. For each parameter group, we report the median value and the "worst-case" diagnostic (Maximum $\hat{R}$ and Minimum ESS) to demonstrate the robustness of the posterior estimates across all 430 sampling locations and 8 habitat strata.

| **Parameter Group** | $n$ | **Median**  $\hat{R}$ | **Max**  $\hat{R}$ | **Median ESS** | **Min ESS** |
| --- | --- | --- | --- | --- | --- |
| Detection ($\beta$) | 8 | 1.00 | 1.00 | 18,004 | 12,008 |
| Detection scale $\left( \sigma\right)$ | 1,929 | 1.00 | 1.00 | 18,177 | 12,026 |
| Abundance $\left( N \right)$ | 1,929 | 1.00 | 1.02 | 22,768 | 5,572 |
| Occasion density ($\gamma_{occ}$) | 1,929 | 1.00 | 1.04 | 20,891 | 281 |
| Section mean density $\left( \mu_{\gamma,s} \right)$ | 430 | 1.00 | 1.04 | 19,423 | 289 |
| Global Variance ($\sigma_{\gamma}$) | 1 | 1.00 | 1.00 | 7,299 | 7,299 |

**Sensitivity Analysis and Comparative Evaluation of Modelling Frameworks**

**Overview**

We conducted a sensitivity analysis comparing the method we used in the manuscript to two established methods. We first fitted a stratified Conventional Distance Sampling (CDS) model using the Distance package (Thomas et al., 2010) and then a series of integrated distance sampling models using the gdistsamp function in the unmarked package (Fiske & Chandler, 2011).

**1. Conventional Distance Sampling (CDS)**

**Model structure**

The model used a stratified Conventional Distance Sampling (CDS) approach in the Distance package. CDS estimates population size in two stages:

1. Detection function estimation
   A detection function describing the probability of detecting an individual as a function of its distance from the transect line is fitted using the observed detection distances.
2. Abundance extrapolation
   Observed counts are then scaled to the entire survey area using the estimated detection probability and the total survey effort.

The model fitted a single half-normal detection function with vegetation type as a covariate and where detection scale varies with vegetation type. Population size was estimated separately for each year × habitat by combining the detection function with the observed counts and sampling effort.

**Key assumptions**

This framework assumes that:

- surveys represent independent snapshots of the population each year
- sampled transects are representative of the entire region
- all individuals are available for detection during the survey

Unlike hierarchical models, CDS does not explicitly model variation in availability or latent abundance.

**Results**

| **Year** | **Estimate** | **LCL** | **UCL** | **Area** |
| --- | --- | --- | --- | --- |
| 2018 | 166,679 | 117,030 | 261,708 | 153 |
| 2019 | 200,988 | 133,008 | 345,684 | 153 |
| 2020 | 171,391 | 111,849 | 284,286 | 153 |
| 2021 | 191,185 | 129,198 | 314,608 | 153 |
| 2022 | 231,683 | 118,467 | 72,578,874 | 148 |

The estimated population sizes are broadly consistent with the Bayesian estimates but confidence intervals are much wider (See Figure SD1).

**Limitations observed**

**Structural limitation:**
The CDS estimator requires survey effort to be defined for every stratum included in the population total. Some habitats (e.g. Champignon) were not surveyed in 2022 meaning that they could not be included in the abundance calculation.

**Parameter instability in rare habitats:**
Habitats with limited sampling effort (e.g. Sand; *n* = 4 transect sections) produced highly uncertain estimates: Sand’s coefficient of variation exceeded 50%.

**Sensitivity to sampling imbalance:**
CDS assumes that surveyed transects provide an unbiased representation of the entire study area. When survey effort is biased or unevenly distributed across habitats or years the estimator is unable to account for this imbalance.

**2. Integrated Distance Sampling (unmarked package)**

To address the limitations of CDS, we next implemented likelihood-based integrated distance sampling models using the gdistsamp function in the unmarked package.

Unlike CDS, these models jointly estimate:

- λ (abundance)
- ϕ (availability)
- p (detection)

within a single likelihood framework.

The models were fitted in three stages of increasing simplification.

**2.1 Saturated Integrated Model**

**Model structure**

The first attempt replicated the structure of the Bayesian model as closely as possible by including section-specific fixed effects for abundance with additional effects of year on availability and vegetation type on detection

This specification requires the estimation of over 400 section-level abundance parameters.

The model failed to converge suggesting that the likelihood could not uniquely estimate the large number of parameters relative to the available data.

**Interpretation**

This failure reflects a common limitation of maximum likelihood estimation when the number of parameters approaches the number of observations. In contrast the Bayesian model accommodates this complexity through hierarchical shrinkage which allows section-level parameters to borrow strength from the overall dataset.

**2.2 Temporal Availability Model**

**Model structure**

To reduce parameter complexity, section-level effects were removed and abundance was instead modelled as a function of vegetation type only and availability was allowed to vary by year. Detection probability continued to vary by vegetation type.

**Results**

| **Year** | **Population estimate** |
| --- | --- |
| 2018 | 101,534 |
| 2019 | 44,793 |
| 2020 | 125,803 |
| 2021 | 55,624 |
| 2022 | 72,499 |

This model converged but produced highly unstable population estimates with results suggesting dramatic year-to-year population fluctuations.

**Interpretation**

These fluctuations arise because the model must separate changes in abundance (λ) and availability (ϕ) using limited information. When sampling effort is uneven these parameters become weakly identifiable which causes the model to attribute variation in sampling coverage to changes in availability resulting in some very low and unlikely population estimates (e.g. 44,759 in 2019 and 55,624 in 2021).

**2.3 Static Integrated Model**

**Model structure**

To stabilize the estimation, availability was assumed to be constant across years while abundance remained a function of vegetation type.

**Result**

This produced a single stable atoll-wide estimate: $N=160, 879$

**Interpretation**

Although this model converged successfully, it imposes the strong assumption that both abundance and availability are constant across all survey years. The population estimate is also substantially lower than both the Bayesian and CDS analysis.

**2.4 Habitat Density Model with Yearly Availability**

We then fitted a generalized distance sampling model in which Habitat-specific density (λ) was assumed constant across years, Availability (ϕ) varied between years and Detection probability (p) varied by vegetation type.

Abundance was estimated as density per square kilometre and then scaled to the total habitat area.

**Results**

| **Year** | **Population estimate** |
| --- | --- |
| 2018 | 186,419 |
| 2019 | 82,238 |
| 2020 | 230,976 |
| 2021 | 102,124 |
| 2022 | 133,106 |

**Interpretation**

This model produced more realistic population magnitudes by estimating density per unit area (km^2^) but still exhibited substantial year-to-year variation driven entirely by the estimated availability parameter. Because habitat densities were fixed across years the model attributes temporal variation in observed counts solely to changes in availability. Without the hierarchical structure the model cannot distinguish between a year where tortoises were unavailable and a year where sampling effort was simply lower or poorly placed.

**2.5 Integrated HDS (Abundance Trend Model)**
Finally, we implemented a model where abundance ($\lambda$) was allowed to vary by year, but availability was assumed constant. This model treats each year as an independent snapshot and is blind to sampling bias.

**Results**

| **Year** | **Population estimate** |
| --- | --- |
| 2018 | 91,345 |
| 2019 | 96,799 |
| 2020 | 92,565 |
| 2021 | 100,793 |
| 2022 | 92,095 |

**Interpretation**

This model achieved greater stability by fixing abundance ($\lambda$) across years which resulted in estimates that fluctuate within a narrow range. The estimates are significantly lower than both the Bayesian and CDS results because the framework fails to hierarchically account for the low detectability in dense scrub habitats and effectively "averaging out" the detection function and missing cryptic individuals. Because habitat densities were fixed, the model is forced to attribute all temporal variation in observed counts to changes in animal availability ($\phi$). This creates a static census that is unable to distinguish between true population trends and annual shifts in tortoise behaviour or sampling effort.

**Figure S8.** Comparison of atoll-wide population trajectories across five modelling frameworks. Shaded ribbons represent 95% Credible Intervals for the Bayesian model and 95% Confidence Intervals for frequentist models. For models 1, 2.2, 2.3, 2.4 and 2.5 uncertainty was propagated into the atoll-wide total using a parametric bootstrap (500 iterations), where coefficients were sampled from the multivariate normal distribution defined by the model’s maximum likelihood estimates and variance-covariance matrix.

The Bayesian hierarchical framework provides a tractable and fully transparent modelling approach in which uncertainty is explicitly propagated through all components of the model. While elements of model behaviour can be inspected in the above approaches (e.g. detection functions in CDS or parameter estimates in integrated distance sampling models) these methods do not estimate latent state variables or provide full posterior distributions. They therefore offer limited ability to diagnose how individual sections, habitats or years contribute to the final population estimates. Estimating posterior distributions for both parameters and latent state variables allows the Bayesian modeller to estimate the full range of plausible abundance, detection and availability values to be examined. The hierarchical structure enables partial pooling across sections and habitats which stabilises estimates where data are sparse. Posterior predictions can also be inspected directly at the section and year level, allowing modellers to uncover the drivers of variation in the population estimates—unusual results can be traced back to the underlying data and sampling structure rather than being treated as unexplained artefacts of the estimation procedure.

**Refernces:**

Brooks, S. P., & Gelman, A. (1998). General Methods for Monitoring Convergence of Iterative Simulations. *Journal of Computational and Graphical Statistics*, *7*(4), 434–455.

Fiske, I., & Chandler, R. (2011). unmarked: An R Package for Fitting Hierarchical Models of Wildlife Occurrence and Abundance. *Journal of Statistical Software*, *43*(10). https://doi.org/10.18637/jss.v043.i10

Thomas, L., Buckland, S. T., Rexstad, E. A., Laake, J. L., Strindberg, S., Hedley, S. L., Bishop, J. R. B., Marques, T. A., & Burnham, K. P. (2010). Distance software: design and analysis of distance sampling surveys for estimating population size. *Journal of Applied Ecology*, *47*(1), 5–14. https://doi.org/10.1111/j.1365-2664.2009.01737.x
